# Recent discoveries in the cycling, growing and aging of the p53 field

**DOI:** 10.18632/aging.100529

**Published:** 2012-12-23

**Authors:** James A. McCubrey, Zoya N. Demidenko

**Affiliations:** ^1^ Department of Microbiology & Immunology, Brody School of Medicine, East Carolina University, NC 27858, USA; ^2^ Department of Cell Stress Biology, Roswell Park Cancer Institute, Buffalo, NY 14263, USA

**Keywords:** cancer, aging, MTOR, TOR, longevity

## Abstract

The P53 gene and it product p53 protein is the most studied tumor suppressor, which was considered as oncogene for two decades until 1990. More than 60 thousand papers on the topic of p53 has been abstracted in Pubmed. What yet could be discovered about its role in cell death, growth arrest and apoptosis, as well as a mediator of the therapeutic effect of anticancer drugs. Still during recent few years even more amazing discoveries have been done. Here we review such topics as suppression of epigenetic silencing of a large number of non-coding RNAs, role of p53 in suppression of the senescence phenotype, inhibition of oncogenic metabolism, protection of normal cells from chemotherapy and even tumor suppression without apoptosis and cell cycle arrest.

Not for the first time in the recent years, but the hero again remains p53. Importantly that it was not from one single discovery but instead from several different discoveries and most were unexpected. Gudkov and co-workers recently reported (also on line first) that p53, a tumor suppressor protein, recently renamed TP53, cooperated with DNA methylation to maintain the silencing of a large portion of the mouse genome. (Leonova KI, Brodsky L, Lipchick B, Pal M, Novototskaya L, Chenchik AA, Sen GC, Komarova EA, Gudkov AV. p53 cooperates with DNA methylation and a suicidal interferon response to maintain epigenetic silencing of repeats and noncoding RNAs. PNAS U S A. Epub 2012 Dec 10.) It was previously known that mammalian genomes contained various classes of interspersed and tandem repeat DNA sequences that were transcriptionally inactive. An essential unanswered question was why they are so many and why are they transcriptionally inactive?

The answer to this question was recently provided by the Gudkov team. The transcription of these sequences was determined to be blocked by p53 in conjunction with DNA methylation. In p53-deficient, but not in p53 wild-type mouse embryonic fibroblasts, treatment with a DNA demethylating agent caused massive transcription of short interspersed nuclear elements. These elements that were transcribed are near-centromeric satellite DNAs consisting of tandem repeats and multiple species of noncoding RNAs. Amazingly, the abundance of these transcripts exceeded the level of beta-actin mRNA by more than 150-fold. Accumulation of these transcripts, was accompanied by a strong, endogenous, apoptosis-inducing type I IFN response. This work was recently discussed in detail [1, 2]. This phenomenon, which Gudkov and co-workers named “TRAIN” (for “transcription of repeats activates interferon”), was observed in spontaneous tumors in two models of cancer-prone mice. The authors proposed that p53 and IFN cooperate to prevent accumulation of cells containing activated repeats and provide a plausible explanation for the deregulation of IFN function frequently observed in tumors. Therefore, p53 and IFN are key for genetic stability and therefore relevant to both tumorigenesis and aging.

This phenomenon may be linked to another discovery about the role of p53 and INF in long-lived and cancer-resistant rodents. Gurbunova et al [3] demonstrated that in the blind mole rat *Spalax*, a small subterranean rodent which is distinguished by its adaptations to life underground, there was a remarkable longevity (with a maximum documented lifespan of 21 years), and resistance to spontaneous cancer induction. Spontaneous tumors have never been observed in these rodents. Cells obtained from blind mole rats proliferated for 7-20 population doublings, after which the cells began secreting IFN-beta, and the cultures underwent necrotic cell death. In another long-lived and cancer-resistant rat model, the release of IFN-beta was determined to result in the sequestration of p53 and Rb-rescued necrotic cell death. The precise link between two discoveries needs to be further elucidated. Noteworthy, IFN-beta is currently undergoing phase I clinical trials in various drug combinations [4].

Next we discuss a third phenomenon published in summer of 2012. It was shown that hypoxia, by inhibiting mTOR in human cells, prevented the development of senescent phenotype in non-dividing but not senescent cells [5]. mTOR is known to drive cell senescence in culture [6-9] and its inhibition extends the lifespan of mice [10-16].

As recently proposed, aging is not caused by accumulation of DNA damage but is driven by signaling pathways such as TOR [13-36]. Aging and age-related diseases are quasi-programs, an aimless continuation of developmental growth. The hyperfunction theory was initiated by the hypothesis that active growth-promoting pathway must drive aging instead of growth, if the cell cycle is blocked [37, 38]. This increases cellular functions, leading to hyperfunction, age-related diseases and malfunctions. This theory, Recently named “the hyperfunction theory”, this point of view is becoming increasingly accepted [17, 39, 40].

Besides rapamycin and other rapalogs, mTOR is inhibited by p53 and hypoxia [5, 7]. Long-lived rats that live underground frequently experience hypoxia. Could hypoxia also contribute to their exceptional longevity? Also, it was known that fibroblasts from long-lived mutant mice exhibit lower mTOR activity after nutrient deprivation or oxidative stress [41].

Two recent papers demonstrated that rapamycin can increase life-span in p53- deficient mice, substituting p53 by rapamycin [42, 43]. This may be due to natural inhibition of mTOR by p53, as suggested recently, so rapamycin could potentially substitute for p53-dependent mTOR inhibition and extend lifespan [44]. p53 may not only initiate cell cycle arrest (a condition suitable for conversion to senescence driven by mTOR), but may also suppress this conversion from arrest to senescence by inhibiting mTOR [7]. The choice between senescence and quiescence/apoptosis may be determined by inhibition of mTOR by p53 [5, 8, 45-50].

But the most unexpected discovery was the tumor suppression observed in the absence of p53-mediated cell-cycle arrest, apoptosis, and senescence [51, 52]. What could this result in? Most scientists remain skeptical. Could this missing tumor-suppression activity be gerosupression by p53 as recently discussed [53]. But still this could be a very unique case in exceptional conditions and special mice.

There were numerous exciting reports increasing the diverse roles of p53 as a tumor suppressor [54-75] emphasizing its functions in apoptosis [76-81] and especially prevention of p53-mediated apoptosis by HIF-1 through a secreted neuronal tyrosinase [82] cell cycle arrest [83-86]. p53 has also been shown to be involved in the inhibition of invasiveness [87], and interact with other genes to suppress cancer [88], as well as suppress p63 to prevent induction of a pro-invasive secretome [89]. Moreover p53 has been shown to regulate telomere function [90] and p53 can suppress telomere-driven tetraploidization [91]. Interesting breakthroughs were in the identification of p53 as inhibitor of metabolism, [58, 92-94] its role in autophagy, [95, 96] it roles in induction of necrosis [97] and other diverse activities [98-110].

In fact, some of metabolic effects of p53 are associated with gerosuppression by p53 [53]. Noteworthy, rapamycin, like p53, may not only suppress oncogenic metabolism but also decrease lactate production by cancer cells [111, 112].

Given that the PI3K/mTOR pathway is activated in both aging [13] and cancer [113-125], aging and cancer share such characteristics as an increased metabolism, anabolic phenotype and other metabolic features [126]. By themselves, aerobic cancer cell and stromal metabolism become therapeutic targets [127]. Additionalpromising cancer-specific targets are glutaminase [128] and PKM2 [126-134]. PKM2 expression is necessary for aerobic glycolysis and cell proliferation *in vivo* [129-134]. Pyruvate kinase M2 regulates glucose metabolism by functioning as a coactivator for hypoxia-inducible factor 1 in cancer cells [129-135]. Cancer cells universally express the M2 isoform of the glycolytic enzyme pyruvate kinase (PKM2). Although isoform selective inhibition of PKM2 with small molecules is feasible and support the hypothesis that inhibition of glucose metabolism in cancer cells is a viable strategy to treat human malignancy [125], the cancer-selectivity of PKM2 was recently doubted [136].

But here is a new twist: p53 may protect cells lacking p53 (all normal cells), thus in theory decreasing side effects, without decreasing the therapeutic effects against cancer cells lacking p53. Thus, it was shown that p53-mediated senescence impairs the apoptotic response to chemotherapy and clinical outcome in breast cancer [137]. But here is a silver edge of the cloud [138]. By inducing cytostatic levels of p53 and causing quiescence, we can protect normal cells from chemotherapy, without protection of cancer cells lacking p53. Protection of normal cells was called cyclotherapy [139-142]. Protection of normal cells by induction of p53 was further confirmed recently [143-147].

Finally, the role of p53 in somatic cell reprogramming was recently discussed in detail [148-153].

## References

[R1] Levine AJ, Greenbaum B (2012). The Maintenance of Epigenetic State of the Epigenome. Oncotarget.

[R2] Van Meter M, Seluanov A, Gorbunova V (2012). Forever young? Exploring the link between rapamycin, longevity and cancer. Cell Cycle.

[R3] Gorbunova V, Hine C, Tian X, Ablaeva J, Gudkov AV, Nevo E, Seluanov A (2012). Cancer resistance in the blind mole rat is mediated by concerted necrotic cell death mechanism. Proc Natl Acad Sci U S A.

[R4] Yi T, Elson P, Mitsuhashi M, Jacobs B, Hollovary E, Budd TG, Spiro T, Triozzi P, Borden EC (2011). Phosphatase inhibitor, sodium stibogluconate, in combination with interferon (IFN) alpha 2b: phase I trials to identify pharmacodynamic and clinical effects. Oncotarget.

[R5] Leontieva OV, Natarajan V, Demidenko ZN, Burdelya LG, Gudkov AV, Blagosklonny MV (2012). Hypoxia suppresses conversion from proliferative arrest to cellular senescence. Proc Natl Acad Sci U S A.

[R6] Demidenko ZN, Blagosklonny MV (2008). Growth stimulation leads to cellular senescence when the cell cycle is blocked. Cell Cycle.

[R7] Demidenko ZN, Korotchkina LG, Gudkov AV, Blagosklonny MV (2010). Paradoxical suppression of cellular senescence by p53. Proc Natl Acad Sci U S A.

[R8] Korotchkina LG, Leontieva OV, Bukreeva EI, Demidenko ZN, Gudkov AV, Blagosklonny MV (2010). The choice between p53-induced senescence and quiescence is determined in part by the mTOR pathway. Aging (Albany NY).

[R9] Leontieva OV, Blagosklonny MV (2010). DNA damaging agents and p53 do not cause senescence in quiescent cells, while consecutive re-activation of mTOR is associated with conversion to senescence. Aging (Albany NY).

[R10] Harrison DE, Strong R, Sharp ZD, Nelson JF, Astle CM, Flurkey K, Nadon NL, Wilkinson JE, Frenkel K, Carter CS, Pahor M, Javors MA, Fernandezr E, Miller RA (2009). Rapamycin fed late in life extends lifespan in genetically heterogenous mice. Nature.

[R11] Miller RA, Harrison DE, Astle CM, Baur JA, Boyd AR, de Cabo R, Fernandez E, Flurkey K, Javors MA, Nelson JF, Orihuela CJ, Pletcher S, Sharp ZD, Sinclair D, Starnes JW, Wilkinson JE (2011). Rapamycin, but not resveratrol or simvastatin, extends life span of genetically heterogeneous mice. J Gerontol A Biol Sci Med Sci.

[R12] Wilkinson JE, Burmeister L, Brooks SV, Chan CC, Friedline S, Harrison DE, Hejtmancik JF, Nadon N, Strong R, Wood LK, Woodward MA, Miller RA (2012). Rapamycin slows aging in mice. Aging Cell.

[R13] Blagosklonny MV (2010). Rapamycin and quasi-programmed aging: Four years later. Cell Cycle.

[R14] Anisimov VN, Zabezhinski MA, Popovich IG, Piskunova TS, Semenchenko AV, Tyndyk ML, Yurova MN, Rosenfeld SV, Blagosklonny MV (2011). Rapamycin increases lifespan and inhibits spontaneous tumorigenesis in inbred female mice. Cell Cycle.

[R15] Anisimov VN, Zabezhinski MA, Popovich IG, Piskunova TS, Semenchenko AV, Tyndyk ML, Yurova MN, Antoch MP, Blagosklonny MV (2010). Rapamycin extends maximal lifespan in cancer-prone mice. Am J Pathol.

[R16] Spong A, Bartke A (2012). Rapamycin slows aging in mice. Cell Cycle.

[R17] Stipp D (2012). A new path to longevity. Sci Am.

[R18] Blagosklonny MV (2006). Aging and immortality: quasi-programmed senescence and its pharmacologic inhibition. Cell Cycle.

[R19] Blagosklonny MV (2010). Linking calorie restriction to longevity through sirtuins and autophagy: any role for TOR. Cell Death Dis.

[R20] Blagosklonny MV (2010). Calorie restriction: Decelerating mTOR-driven aging from cells to organisms (including humans). Cell Cycle.

[R21] Blagosklonny MV (2010). Why men age faster but reproduce longer than women: mTOR and evolutionary perspectives. Aging (Albany NY).

[R22] Blagosklonny MV (2010). Increasing healthy lifespan by suppressing aging in our lifetime: Preliminary proposal. Cell Cycle.

[R23] Blagosklonny MV (2010). Revisiting the antagonistic pleiotropy theory of aging: TOR-driven program and quasi-program. Cell Cycle.

[R24] Blagosklonny MV (2010). Why human lifespan is rapidly increasing: solving “longevity riddle” with “revealed-slow-aging” hypothesis. Aging (Albany NY).

[R25] Blagosklonny MV (2010). Why the disposable soma theory cannot explain why women live longer and why we age. Aging (Albany NY).

[R26] Blagosklonny MV (2011). Hormesis does not make sense except in the light of TOR-driven aging. Aging (Albany NY).

[R27] Blagosklonny MV (2011). Molecular damage in cancer: an argument for mTOR-driven aging. Aging (Albany NY).

[R28] Blagosklonny MV (2011). Rapamycin-induced glucose intolerance: Hunger or starvation diabetes. Cell Cycle.

[R29] Blagosklonny MV (2012). Cell cycle arrest is not yet senescence, which is not just cell cycle arrest: terminology for TOR-driven aging. Aging (Albany NY).

[R30] Blagosklonny MV (2012). Prospective treatment of age-related diseases by slowing down aging. Am J Pathol.

[R31] Kaeberlein M, Powers RWr, Steffen KK, Westman EA, Hu D, Dang N, Kerr EO, Kirkland KT, Fields S, Kennedy BK (2005). Regulation of yeast replicative life span by TOR and Sch9 in response to nutrients. Science.

[R32] Ayyadevara S, Alla R, Thaden JJ, Shmookler Reis RJ (2008). Remarkable longevity and stress resistance of nematode PI3K-null mutants. Aging Cell.

[R33] Stanfel MN, Shamieh LS, Kaeberlein M, Kennedy BK (2009). The TOR pathway comes of age. Biochim Biophys Acta.

[R34] Selman C, Lingard S, Choudhury AI, Batterham RL, Claret M, Clements M, Ramadani F, Okkenhaug K, Schuster E, Blanc E, Piper MD, Al-Qassab H, Speakman J.R, Carmignac D, Robinson I.C, Thornton J.M, Gems D, Partridge L, Withers D.J (2008). Evidence for lifespan extension and delayed age-related biomarkers in insulin receptor substrate 1 null mice. FASEB J.

[R35] Selman C, Tullet JM, Wieser D, Irvine E, Lingard SJ, Choudhury AI, Claret M, Al-Qassab H, Carmignac D, Ramadani F, Woods A, Robinson IC, Schuster E, Batterham RL, Kozma SC, Thomas G (2009). Ribosomal protein S6 kinase 1 signaling regulates mammalian life span. Science.

[R36] Kapahi P, Chen D, Rogers AN, Katewa SD, Li PW, Thomas EL, Kockel L (2010). With TOR, less is more: a key role for the conserved nutrient-sensing TOR pathway in aging. Cell Metab.

[R37] Blagosklonny MV (2003). Cell senescence and hypermitogenic arrest. EMBO Rep.

[R38] Blagosklonny MV (2011). Cell cycle arrest is not senescence. Aging (Albany NY).

[R39] Gems DH, de la Guardia YI (2012). Alternative Perspectives on Aging in C. elegans: Reactive Oxygen Species or Hyperfunction?. Antioxid Redox Signal.

[R40] Gems D, Partridge L (2012). Genetics of Longevity in Model Organisms: Debates and Paradigm Shifts. Annu Rev Physiol.

[R41] Wang M, Miller RA (2012). Fibroblasts from long-lived mutant mice exhibit increased autophagy and lower TOR activity after nutrient deprivation or oxidative stress. Aging Cell.

[R42] Komarova EA, Antoch MP, Novototskaya LR, Chernova OB, Paszkiewicz G, Leontieva OV, Blagosklonny MV, Gudkov AV (2012). Rapamycin extends lifespan and delays tumorigenesis in heterozygous p53+/− mice. Aging (Albany NY).

[R43] Comas M, Toshkov I, Kuropatwinski KK, Chernova OB, Polinsky A, Blagosklonny MV, Gudkov AV, Antoch MP (2012). New nanoformulation of rapamycin Rapatar extends lifespan in homozygous p53−/− mice by delaying carcinogenesis. Aging (Albany NY).

[R44] Blagosklonny MV (2012). Rapalogs in cancer prevention: Anti-aging or anticancer?. Cancer Biol Ther.

[R45] Serrano M (2010). Shifting senescence into quiescence by turning up p53. Cell Cycle.

[R46] Leontieva O, Gudkov A, Blagosklonny M (2010). Weak p53 permits senescence during cell cycle arrest. Cell Cycle.

[R47] Leontieva OV, Demidenko ZN, Gudkov AV, Blagosklonny MV (2011). Elimination of proliferating cells unmasks the shift from senescence to quiescence caused by rapamycin. PLoS One.

[R48] Galluzzi L, Kepp O, Kroemer G (2010). TP53 and MTOR crosstalk to regulate cellular senescence. Aging (Albany NY).

[R49] Lane DP, Verma C, Fang CC (2010). The p53 inducing drug dosage may determine quiescence or senescence. Aging (Albany NY).

[R50] Maki CG (2010). Decision-making by p53 and mTOR. Aging (Albany NY).

[R51] Li T, Kon N, Jiang L, Tan M, Ludwig T, Zhao Y, Baer R, Gu W (2012). Tumor suppression in the absence of p53-mediated cell-cycle arrest, apoptosis, and senescence. Cell.

[R52] Hock AK, Vousden KH (2012). Tumor suppression by p53: fall of the triumvirate?. Cell.

[R53] Blagosklonny MV (2012). Tumor suppression by p53 without apoptosis and senescence: conundrum or rapalog-like gerosuppression?. Aging (Albany NY).

[R54] Feldser DM, Kostova KK, Winslow MM, Taylor SE, Cashman C, Whittaker CA, Sanchez-Rivera FJ, Resnick R, Bronson R, Hemann MT, Jacks T (2011). Stage-specific sensitivity to p53 restoration during lung cancer progression. Nature.

[R55] Knappskog S, Lonning PE (2011). MDM2 promoter SNP285 and SNP309; phylogeny and impact on cancer risk. Oncotarget.

[R56] Schlereth K, Charles JP, Bretz AC, Stiewe T (2010). Life or death: p53-induced apoptosis requires DNA binding cooperativity. Cell Cycle.

[R57] Bao W, Chen M, Zhao X, Kumar R, Spinnler C, Thullberg M, Issaeva N, Selivanova G, Stromblad S (2011). PRIMA-1Met/APR-246 induces wild-type p53-dependent suppression of malignant melanoma tumor growth in 3D culture and in vivo. Cell Cycle.

[R58] Madan E, Gogna R, Bhatt M, Pati U, Kuppusamy P, Mahdi AA (2011). Regulation of glucose metabolism by p53: emerging new roles for the tumor suppressor. Oncotarget.

[R59] Junttila MR, Karnezis AN, Garcia D, Madriles F, Kortlever RM, Rostker F, Brown Swigart L, Pham DM, Seo Y, Evan GI, Martins CP (2010). Selective activation of p53-mediated tumour suppression in high-grade tumours. Nature.

[R60] Madar S, Stambolsky P, Rotter V (2011). Unleash the wild type: restoration of p53 suppressive activity in skin cancer. Cell Cycle.

[R61] Stegh AH, DePinho RA (2011). Beyond effector caspase inhibition: Bcl2L12 neutralizes p53 signaling in glioblastoma. Cell Cycle.

[R62] Carr-Wilkinson J, Griffiths R, Elston R, Gamble LD, Goranov B, Redfern CP, Lunec J, Tweddle DA (2011). Outcome of the p53-mediated DNA damage response in neuroblastoma is determined by morphological subtype and MYCN expression. Cell Cycle.

[R63] Li L, Wang L, Wang Z, Ho Y, McDonald T, Holyoake TL, Chen W, Bhatia R (2012). Activation of p53 by SIRT1 inhibition enhances elimination of CML leukemia stem cells in combination with imatinib. Cancer Cell.

[R64] Botcheva K, McCorkle SR, McCombie WR, Dunn JJ, Anderson CW (2011). Distinct p53 genomic binding patterns in normal and cancer-derived human cells. Cell Cycle.

[R65] Miliani de Marval PL, Zhang Y (2011). The RP-Mdm2-p53 pathway and tumorigenesis. Oncotarget.

[R66] Chow LM, Endersby R, Zhu X, Rankin S, Qu C, Zhang J, Broniscer A, Ellison DW, Baker SJ (2011). Cooperativity within and among Pten, p53, and Rb pathways induces high-grade astrocytoma in adult brain. Cancer Cell.

[R67] Roe JS, Kim HR, Hwang IY, Ha NC, Kim ST, Cho EJ, Youn HD (2011). Phosphorylation of von Hippel-Lindau protein by checkpoint kinase 2 regulates p53 transactivation. Cell Cycle.

[R68] Jiang Z, Jones R, Liu JC, Deng T, Robinson T, Chung PE, Wang S, Herschkowitz JI, Egan SE, Perou CM, Zacksenhaus E (2011). RB1 and p53 at the crossroad of EMT and triple-negative breast cancer. Cell Cycle.

[R69] Yu X, Vazquez A, Levine AJ, Carpizo DR (2012). Allele-specific p53 mutant reactivation. Cancer Cell.

[R70] Bywater MJ, Poortinga G, Sanij E, Hein N, Peck A, Cullinane C, Wall M, Cluse L, Drygin D, Anderes K, Huser N, Proffitt C, Bliesath J, Haddach M, Schwaebe MK, Ryckman DM (2012). Inhibition of RNA polymerase I as a therapeutic strategy to promote cancer-specific activation of p53. Cancer Cell.

[R71] Baudot AD, Ryan KM (2011). p53 and tumor surveillance: killer finds way to recruit assassins. Cell Cycle.

[R72] Llanos S, Serrano M (2010). Depletion of ribosomal protein L37 occurs in response to DNA damage and activates p53 through the L11/MDM2 pathway. Cell Cycle.

[R73] Li B, Cheng Q, Li Z, Chen J (2010). p53 inactivation by MDM2 and MDMX negative feedback loops in testicular germ cell tumors. Cell Cycle.

[R74] O'Prey J, Crighton D, Martin AG, Vousden KH, Fearnhead HO, Ryan KM (2010). p53-mediated induction of Noxa and p53AIP1 requires NFkappaB. Cell Cycle.

[R75] Cipriano R, Patton JT, Mayo LD, Jackson MW (2010). Inactivation of p53 signaling by p73 or PTEN ablation results in a transformed phenotype that remains susceptible to Nutlin-3 mediated apoptosis. Cell Cycle.

[R76] Nardinocchi L, Puca R, D'Orazi G (2011). HIF-1alpha antagonizes p53-mediated apoptosis by triggering HIPK2 degradation. Aging (Albany NY).

[R77] Hill R, Madureira PA, Waisman DM, Lee PW (2011). DNA-PKCS binding to p53 on the p21WAF1/CIP1 promoter blocks transcription resulting in cell death. Oncotarget.

[R78] Azzam GA, Frank AK, Hollstein M, Murphy ME Tissue-specific apoptotic effects of the p53 codon 72 polymorphism in a mouse model. Cell Cycle.

[R79] Trinh DL, Elwi AN, Kim SW (2010). Direct interaction between p53 and Tid1 proteins affects p53 mitochondrial localization and apoptosis. Oncotarget.

[R80] He J, Gu L, Zhang H, Zhou M (2011). Crosstalk between MYCN and MDM2-p53 signal pathways regulates tumor cell growth and apoptosis in neuroblastoma. Cell Cycle.

[R81] Zhao CY, Grinkevich VV, Nikulenkov F, Bao W, Selivanova G (2011). Rescue of the apoptotic-inducing function of mutant p53 by small molecule RITA. Cell Cycle.

[R82] Sendoel A, Kohler I, Fellmann C, Lowe SW, Hengartner MO (2010). HIF-1 antagonizes p53-mediated apoptosis through a secreted neuronal tyrosinase. Nature.

[R83] Mallette FA, Calabrese V, Ilangumaran S, Ferbeyre G (2010). SOCS1, a novel interaction partner of p53 controlling oncogene-induced senescence. Aging (Albany NY).

[R84] Landsverk KS, Patzke S, Rein ID, Stokke C, Lyng H, De Angelis PM, Stokke T Three independent mechanisms for arrest in G2 after ionizing radiation. Cell Cycle.

[R85] Tavana O, Benjamin CL, Puebla-Osorio N, Sang M, Ullrich SE, Ananthaswamy HN, Zhu C (2010). Absence of p53-dependent apoptosis leads to UV radiation hypersensitivity, enhanced immuno-suppression and cellular senescence. Cell Cycle.

[R86] Jee HJ, Kim AJ, Song N, Kim HJ, Kim M, Koh H, Yun J (2010). Nek6 overexpression antagonizes p53-induced senescence in human cancer cells. Cell Cycle.

[R87] Elyada E, Pribluda A, Goldstein RE, Morgenstern Y, Brachya G, Cojocaru G, Snir-Alkalay I, Burstain I, Haffner-Krausz R, Jung S, Wiener Z, Alitalo K, Oren M, Pikarsky E, Ben-Neriah Y (2011). CKIalpha ablation highlights a critical role for p53 in invasiveness control. Nature.

[R88] Krimpenfort P, Song JY, Proost N, Zevenhoven J, Jonkers J, Berns A (2012). Deleted in colorectal carcinoma suppresses metastasis in p53-deficient mammary tumours. Nature.

[R89] Neilsen PM, Noll JE, Suetani RJ, Schulz RB, Al-Ejeh F, Evdokiou A, Lane DP, Callen DF (2011). Mutant p53 uses p63 as a molecular chaperone to alter gene expression and induce a pro-invasive secretome. Oncotarget.

[R90] Horikawa I, Fujita K, Harris CC (2011). p53 governs telomere regulation feedback too, via TRF2. Aging (Albany NY).

[R91] Davoli T, de Lange T (2012). Telomere-driven tetraploidization occurs in human cells undergoing crisis and promotes transformation of mouse cells. Cancer Cell.

[R92] Vigneron A, Vousden KH (2010). p53, ROS and senescence in the control of aging. Aging (Albany NY).

[R93] Ashur-Fabian O, Har-Zahav A, Shaish A, Wiener Amram H, Margalit O, Weizer-Stern O, Dominissini D, Harats D, Amariglio N, Rechavi G (2010). apoB and apobec1, two genes key to lipid metabolism, are transcriptionally regulated by p53. Cell Cycle.

[R94] Freed-Pastor WA, Mizuno H, Zhao X, Langerod A, Moon SH, Rodriguez-Barrueco R, Barsotti A, Chicas A, Li W, Polotskaia A, Bissell MJ, Osborne TF, Tian B, Lowe SW, Silva JM, Borresen-Dale AL (2012). Mutant p53 disrupts mammary tissue architecture via the mevalonate pathway. Cell.

[R95] Lee IH, Kawai Y, Fergusson MM, Rovira II, Bishop AJ, Motoyama N, Cao L, Finkel T (2012). Atg7 modulates p53 activity to regulate cell cycle and survival during metabolic stress. Science.

[R96] Livesey KM, Kang R, Vernon P, Buchser W, Loughran P, Watkins SC, Zhang L, Manfredi JJ, Zeh HJ, Li L, Lotze MT, Tang D (2012). p53/HMGB1 complexes regulate autophagy and apoptosis. Cancer Res.

[R97] Vaseva AV, Marchenko ND, Ji K, Tsirka SE, Holzmann S, Moll UM (2012). p53 opens the mitochondrial permeability transition pore to trigger necrosis. Cell.

[R98] Leonova KI, Shneyder J, Antoch MP, Toshkov IA, Novototskaya LR, Komarov PG, Komarova EA, Gudkov AV (2010). A small molecule inhibitor of p53 stimulates amplification of hematopoietic stem cells but does not promote tumor development in mice. Cell Cycle.

[R99] Antico Arciuch VG, Russo MA, Dima M, Kang KS, Dasrath F, Liao XH, Refetoff S, Montagna C, Di Cristofano A (2011). Thyrocyte-specific inactivation of p53 and Pten results in anaplastic thyroid carcinomas faithfully recapitulating human tumors. Oncotarget.

[R100] Purvis JE, Karhohs KW, Mock C, Batchelor E, Loewer A, Lahav G (2012). p53 dynamics control cell fate. Science.

[R101] Patel BB, Li XM, Dixon MP, Blagoi EL, Nicolas E, Seeholzer SH, Cheng D, He YA, Coudry RA, Howard SD, Riddle DM, Cooper HC, Boman BM, Conrad P, Crowell JA, Bellacosa A (2011). APC +/− alters colonic fibroblast proteome in FAP. Oncotarget.

[R102] Golomb L, Oren M (2011). DePICTing p53 activation: a new nucleolar link to cancer. Cancer Cell.

[R103] Galluzzi L, Morselli E, Kepp O, Maiuri MC, Kroemer G (2010). Defective autophagy control by the p53 rheostat in cancer. Cell Cycle.

[R104] Rausch T, Jones DT, Zapatka M, Stutz AM, Zichner T, Weischenfeldt J, Jager N, Remke M, Shih D, Northcott PA, Pfaff E, Tica J, Wang Q, Massimi L, Witt H, Bender S Genome sequencing of pediatric medulloblastoma links catastrophic DNA rearrangements with TP53 mutations. Cell.

[R105] Li H, Lakshmikanth T, Garofalo C, Enge M, Spinnler C, Anichini A, Szekely L, Karre K, Carbone E, Selivanova G (2011). Pharmacological activation of p53 triggers anticancer innate immune response through induction of ULBP2. Cell Cycle.

[R106] Brady CA, Jiang D, Mello SS, Johnson TM, Jarvis LA, Kozak MM, Kenzelmann Broz D, Basak S, Park EJ, McLaughlin ME, Karnezis AN, Attardi LD (2011). Distinct p53 transcriptional programs dictate acute DNA-damage responses and tumor suppression. Cell.

[R107] Lu WJ, Chapo J, Roig I, Abrams JM (2010). Meiotic recombination provokes functional activation of the p53 regulatory network. Science.

[R108] Hwang CI, Choi J, Zhou Z, Flesken-Nikitin A, Tarakhovsky A, Nikitin AY (2011). MET-dependent cancer invasion may be preprogrammed by early alterations of p53-regulated feedforward loop and triggered by stromal cell-derived HGF. Cell Cycle.

[R109] Morselli E, Shen S, Ruckenstuhl C, Bauer MA, Marino G, Galluzzi L, Criollo A, Michaud M, Maiuri MC, Chano T, Madeo F, Kroemer G (2011). p53 inhibits autophagy by interacting with the human ortholog of yeast Atg17, RB1CC1/FIP200. Cell Cycle.

[R110] Sarig R, Tzahor E (2011). p53 and epithelial-mesenchymal transition: a linking thread between embryogenesis and cancer. Cell Cycle.

[R111] Ide T, Chu K, Aaronson SA, Lee SW (2010). GAMT joins the p53 network: branching into metabolism. Cell Cycle.

[R112] Leontieva OV, Blagosklonny MV (2011). Yeast-like chronological senescence in mammalian cells: phenomenon, mechanism and pharmacological suppression. Aging (Albany NY).

[R113] Schmidt-Kittler O, Zhu J, Yang J, Liu G, Hendricks W, Lengauer C, Gabelli SB, Kinzler KW, Vogelstein B, Huso DL, Zhou S (2010). PI3Kalpha inhibitors that inhibit metastasis. Oncotarget.

[R114] Weber GL, Parat MO, Binder ZA, Gallia GL, Riggins GJ (2011). Abrogation of PIK3CA or PIK3R1 reduces proliferation, migration, and invasion in glioblastoma multiforme cells. Oncotarget.

[R115] Gruppuso PA, Boylan JM, Sanders JA (2011). The physiology and pathophysiology of rapamycin resistance: implications for cancer. Cell Cycle.

[R116] Bhatia B, Nahle Z, Kenney AM (2010). Double trouble: when sonic hedgehog signaling meets TSC inactivation. Cell Cycle.

[R117] Ericson K, Gan C, Cheong I, Rago C, Samuels Y, Velculescu VE, Kinzler KW, Huso DL, Vogelstein B, Papadopoulos N (2010). Genetic inactivation of AKT1, AKT2, and PDPK1 in human colorectal cancer cells clarifies their roles in tumor growth regulation. Proc Natl Acad Sci U S A.

[R118] Emerling BM, Akcakanat A (2011). Targeting PI3K/mTOR signaling in cancer. Cancer Res.

[R119] Adams JR, Schachter NF, Liu JC, Zacksenhaus E, Egan SE (2011). Elevated PI3K signaling drives multiple breast cancer subtypes. Oncotarget.

[R120] Garrett JT, Chakrabarty A, Arteaga CL (2011). Will PI3K pathway inhibitors be effective as single agents in patients with cancer?. Oncotarget.

[R121] Feng Z, Zhang H, Levine AJ, Jin S (2005). The coordinate regulation of the p53 and mTOR pathways in cells. Proc Natl Acad Sci U S A.

[R122] Steelman LS, Stadelman KM, Chappell WH, Horn S, Bäsecke J, Cervello M, Nicoletti F, Libra M, Stivala F, Martelli AM, McCubrey JA (2008). Akt as a therapeutic target in cancer. Expert Opin Ther Targets.

[R123] Sokolosky ML, Stadelman KM, Chappell WH, Abrams SL, Martelli AM, Stivala F, Libra M, Nicoletti F, Drobot LB, Franklin RA, Steelman LS, McCubrey JA (2011). Involvement of Akt-1 and mTOR in sensitivity of breast cancer to targeted therapy. Oncotarget.

[R124] Hart JR, Vogt PK (2011). Phosphorylation of AKT: a mutational analysis. Oncotarget.

[R125] Chappell WH, Steelman LS, Long JM, Kempf RC, Abrams SL, Franklin RA, Basecke J, Stivala F, Donia M, Fagone P, Malaponte G, Mazzarino MC, Nicoletti F, Libra M, Maksimovic-Ivanic D, Mijatovic S (2011). Ras/Raf/MEK/ERK and PI3K/PTEN/Akt/mTOR inhibitors: rationale and importance to inhibiting these pathways in human health. Oncotarget.

[R126] Darnell JE (2010). STAT3, HIF-1, glucose addiction and Warburg effect. Aging (Albany NY).

[R127] Wolf A, Agnihotri S, Guha A (2010). Targeting metabolic remodeling in glioblastoma multiforme. Oncotarget.

[R128] Erickson JW, Cerione RA (2010). Glutaminase: a hot spot for regulation of cancer cell metabolism?. Oncotarget.

[R129] Vander Heiden MG, Christofk HR, Schuman E, Subtelny AO, Sharfi H, Harlow EE, Xian J, Cantley LC (2010). Identification of small molecule inhibitors of pyruvate kinase M2. Biochem Pharmacol.

[R130] Christofk HR, Vander Heiden MG, Wu N, Asara JM, Cantley LC (2008). Pyruvate kinase M2 is a phosphotyrosine-binding protein. Nature.

[R131] Hitosugi T, Kang S, Vander Heiden MG, Chung TW, Elf S, Lythgoe K, Dong S, Lonial S, Wang X, Chen GZ, Xie J, Gu TL, Polakiewicz RD, Roesel JL, Boggon TJ, Khuri FR (2009). Tyrosine phosphorylation inhibits PKM2 to promote the Warburg effect and tumor growth. Sci Signal.

[R132] Vander Heiden MG, Christofk HR, Schuman E, Subtelny AO, Sharfi H, Harlow EE, Xian J, Cantley LC Identification of small molecule inhibitors of pyruvate kinase M2. Biochem Pharmacol.

[R133] Anastasiou D, Yu Y, Israelsen WJ, Jiang JK, Boxer MB, Hong BS, Tempel W, Dimov S, Shen M, Jha A, Yang H, Mattaini KR, Metallo CM, Fiske BP, Courtney KD, Malstrom S (2012). Pyruvate kinase M2 activators promote tetramer formation and suppress tumorigenesis. Nat Chem Biol.

[R134] Yang W, Zheng Y, Xia Y, Ji H, Chen X, Guo F, Lyssiotis CA, Aldape K, Cantley LC, Lu Z (2012). ERK1/2-dependent phosphorylation and nuclear translocation of PKM2 promotes the Warburg effect. Nat Cell Biol.

[R135] Luo W, Semenza GL (2011). Pyruvate kinase M2 regulates glucose metabolism by functioning as a coactivator for hypoxia-inducible factor 1 in cancer cells. Oncotarget.

[R136] Bluemlein K, Gruning NM, Feichtinger RG, Lehrach H, Kofler B, Ralser M (2011). No evidence for a shift in pyruvate kinase PKM1 to PKM2 expression during tumorigenesis. Oncotarget.

[R137] Jackson JG, Pant V, Li Q, Chang LL, Quintas-Cardama A, Garza D, Tavana O, Yang P, Manshouri T, Li Y, El-Naggar AK, Lozano G (2012). p53-mediated senescence impairs the apoptotic response to chemotherapy and clinical outcome in breast cancer. Cancer Cell.

[R138] Blagosklonny MV (2012). Wt p53 impairs response to chemotherapy: make lemonade to spare normal cells. Oncotarget.

[R139] Blagosklonny MV, Robey R, Bates S, Fojo T (2000). Pretreatment with DNA-damaging agents permits selective killing of checkpoint-deficient cells by microtubule-active drugs. J Clin Invest.

[R140] Blagosklonny MV, Darzynkiewicz Z (2002). Cyclotherapy: protection of normal cells and unshielding of cancer cells. Cell Cycle.

[R141] Carvajal D, Tovar C, Yang H, Vu BT, Heimbrook DC, Vassilev LT (2005). Activation of p53 by MDM2 antagonists can protect proliferating cells from mitotic inhibitors. Cancer Res.

[R142] Choong ML, Yang H, Lee MA, Lane DP (2009). Specific activation of the p53 pathway by low dose actinomycin D: a new route to p53 based cyclotherapy. Cell Cycle.

[R143] Apontes P, Leontieva OV, Demidenko ZN, Li F, Blagosklonny MV (2011). Exploring long-term protection of normal human fibroblasts and epithelial cells from chemotherapy in cell culture. Oncotarget.

[R144] van Leeuwen IM, Lain S (2011). Pharmacological manipulation of the cell cycle and metabolism to protect normal tissues against conventional anticancer drugs. Oncotarget.

[R145] Steelman LS, Martelli AM, Nicoletti F, McCubrey JA (2011). Exploiting p53 status to enhance effectiveness of chemotherapy by lowering associated toxicity. Oncotarget.

[R146] Rao B, van Leeuwen IM, Higgins M, Campbel J, Thompson AM, Lane DP, Lain S (2010). Evaluation of an Actinomycin D/VX-680 aurora kinase inhibitor combination in p53-based cyclotherapy. Oncotarget.

[R147] Blagosklonny MV The power of chemotherapeutic engineering: arresting cell cycle and suppressing senescence to protect from mitotic inhibitors. Cell Cycle.

[R148] Yi L, Lu C, Hu W, Sun Y, Levine AJ (2012). Multiple roles of p53-related pathways in somatic cell reprogramming and stem cell differentiation. Cancer Res.

[R149] Saunders LR, Sharma AD, Tawney J, Nakagawa M, Okita K, Yamanaka S, Willenbring H, Verdin E (2010). miRNAs regulate SIRT1 expression during mouse embryonic stem cell differentiation and in adult mouse tissues. Aging (Albany NY).

[R150] Bonizzi G, Cicalese A, Insinga A, Pelicci PG (2012). The emerging role of p53 in stem cells. Trends Mol Med.

[R151] Menendez S, Camus S, Izpisua Belmonte JC (2010). p53: guardian of reprogramming. Cell Cycle.

[R152] Zhao J, Pei G (2011). Why cell reprogramming is functionally linked to aging?. Aging (Albany NY).

